# Reactive Oxygen Species: From Harmful Molecules to Fine-Tuning Regulators of Stem Cell Niche Maintenance

**DOI:** 10.1371/journal.pgen.1006251

**Published:** 2016-09-01

**Authors:** Juan Carlos del Pozo

**Affiliations:** Centro de Biotecnologia y Genomica de Plantas (UPM-INIA), Universidad Politecnica de Madrid-Instituto Nacional de Investigación y Tecnología Agraria y Alimentaria, Madrid, Spain; Duke, UNITED STATES

For a long time, reactive oxygen species (ROS) have been considered harmful molecules produced in response to abiotic stress or pathogen attack. Nowadays, it is well accepted that ROS also act as signaling molecules involved in the regulation of physiological processes. In animals, ROS control of cell division and stem cell maintenance is emerging as a hot topic [1]. Recent discoveries have highlighted the importance of ROS in the balance between cell self-renewal and differentiation, which is essential for the proper function of stem cells [2]. For example, in animals, hematopoietic stem cells (HSC)—which mostly reside in a quiescent state in the bone marrow—require low levels of ROS to maintain stem cell self-renewal. Conversely, to induce stem cell differentiation, HSC necessitate high ROS levels, which can be generated by stress or inflammation [3].

In plants, however, our knowledge on ROS function in stem cell niche maintenance is much more limited. In maize, ROS is preferentially produced in the quiescence center (QC), suggesting that a ROS-oxidizing environment is needed to maintain a quiescent state [4]. In *Arabidopsis thaliana*, two of the main ROS molecules, superoxide (O_2_^‾^) and hydrogen peroxide (H_2_O_2_), are differentially distributed in root meristems [5]. This differential ROS distribution controls the transition between cell proliferation and differentiation [6]. In this issue of *PLOS Genetics*, Yu et al. [7] report a role for ROS in maintaining the stem cell niche in Arabidopsis roots. These authors identified the *app1* mutant whose phenotype is an inability to maintain the stem cell niche. *APP1* encodes a mitochondria-localized P-loop NTPase that hydrolyzes ATP in vitro and is involved in generating ROS. Loss-of-function alleles of *APP1* had lower levels of ROS (both O_2_^‾^ and H_2_O_2_) in the root meristem and higher expression of two peroxidases genes, *PER11* and *PER55*, which are involved in ROS detoxification. Although *app1* has similar root meristem size and root growth as the wild type, these mutants displayed higher cell division rates of QC cells and premature distal stem cell (DSC) differentiation. To determine if this phenotype was the consequence of having lower ROS, the authors showed that an increase of O_2_^‾^ or H_2_O_2_ levels by pharmacological treatment was sufficient to rescue the *app1* phenotype. They additionally analyzed the role of ROS in stem cell maintenance by altering ROS levels in wild-type roots. Lowering ROS levels in roots increased both QC cell division and DSC differentiation. Similar phenotypes were obtained by overexpressing *APP1* or by treating wild-type plants with H_2_O_2_, indicating that a correct level of ROS is essential to regulate QC and DSC activity.

To understand how ROS regulate these processes at the molecular level, the authors investigated the expression of different genes involved in maintaining QC status, stem cell activity, or cell division. They analyzed the level of two key transcription factors (TFs) needed to define root distal stem cell niche, *SCARECROW* (*SCR*) and *SHORT ROOT* (*SHR*). Both *SCR* and *SHR* were down-regulated in *app1* mutants and in wild-type roots with reduced levels of H_2_O_2_ and O_2_^‾^, suggesting that ROS levels control the expression of these TFs. The phytohormone auxin has been implicated in maintaining stem cell activity [8] as well as distal stem cell differentiation [9]. However, the authors did not find any alteration of the PLT1 or PLT2 gradient, which are involved in maintaining meristematic activity, or the auxin response marker DR5:GFP in *app1* mutants or in the wild type with lower ROS levels. Based on these data, the authors suggest that APP1-dependent ROS signaling acts independently of the auxin pathway. However, when ROS levels were increased in wild type, the levels of PLT1 and PLT2 gradients were significantly reduced, but *SCR* and *SHR* expression were not affected.

UPBEAT1 is a transcription factor that regulates the expression of a set of peroxidases, which are involved in establishing a gradient of ROS (H_2_O_2_ and O_2_^‾^) distribution in the root meristem. This differential ROS distribution controls the transition from cell proliferation to differentiation and subsequently the meristem size. As *APP1* is expressed in the entire root meristem, how is it possible that APP-dependent ROS only affects the QC and DSC and not the entire meristem? A simple explanation might be that the QC and DSC are highly sensitive to slight variations in ROS levels, while meristematic cells can buffer ROS alterations without affecting their cell division potential. Nevertheless, further experiments are needed to clarify this point.

**Fig 1:**
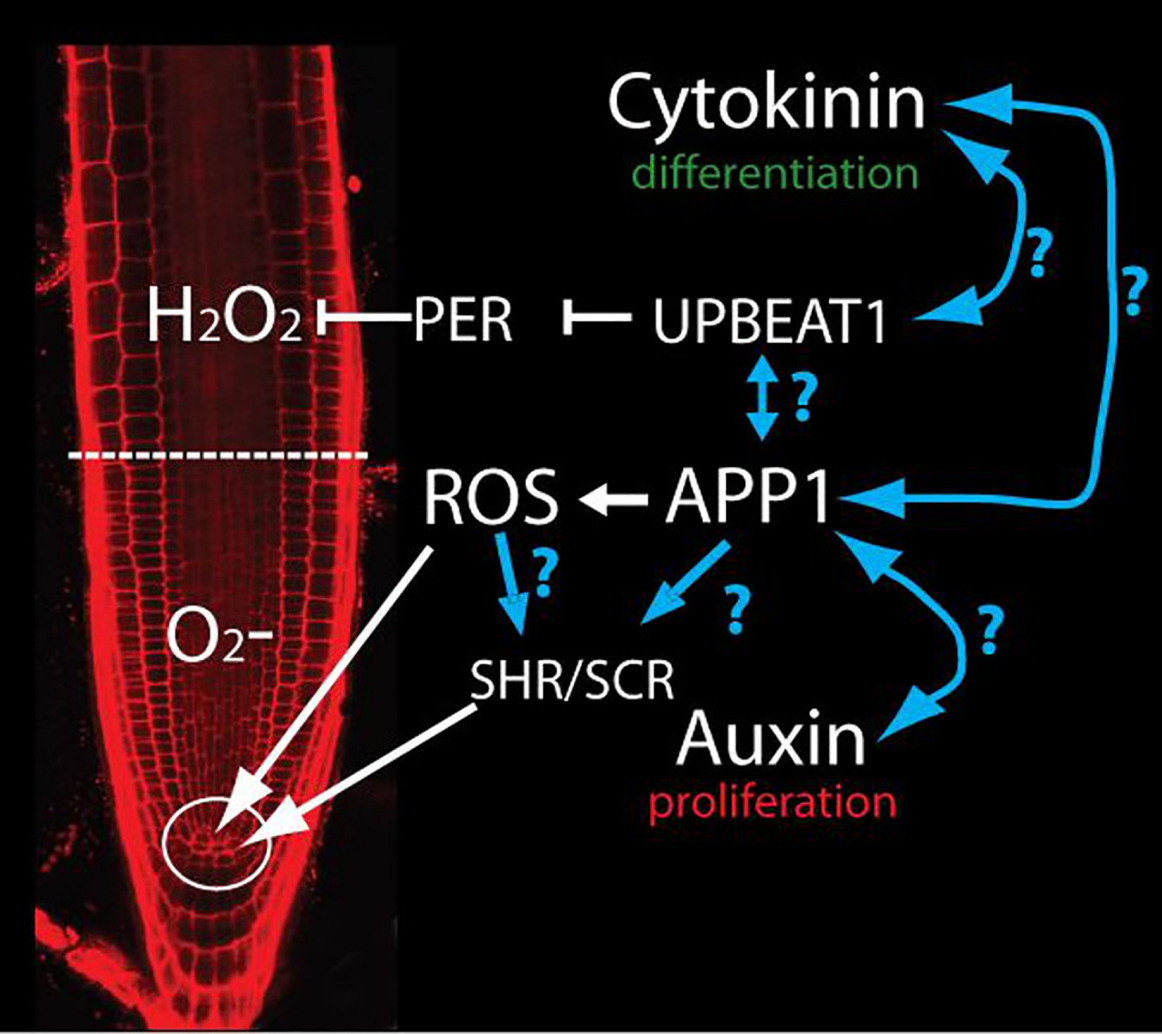
APP1-dependent ROS control QC and DSC through SHR and SCR. APP1 activity generates ROS in mitochondria. These ROS control cell division in QC and DSC in the stem cell niche (circle). UBPEAT1 controls the expression of a set of peroxidases that form an O_2_^‾^/H_2_O_2_-gradient to establish the boundary between cell proliferation and differentiation (dashed line). Auxin and cytokinin regulate cell proliferation and differentiation respectively. Blue lines indicate the possible connections between different pathways that should be tested in the future.

What is next: is ROS activity really disconnected from hormonal signaling in the control of QC and stem cell maintenance in the root meristem? The balance between cell division and differentiation is tightly regulated by the function of several phytohormones and by ROS. This balance is not controlled by a single-hormone signal; rather, it is controlled by the crosstalk between several hormonal pathways. In this work, the authors suggest that the control of stem cell niche maintenance by APP1 is not connected with either auxin signaling or UPBEAT1 function (Fig 1).

However, there are several pieces of evidence that suggest a connection between ROS and hormone signaling. ROS generated in mitochondria by abscisic acid regulates root meristem activity by controlling *PLT* transcription and auxin accumulation in the root tip [10]. Likewise, ROS generated by mitochondrial perturbation has been associated with a reduction in auxin signaling [11]. Recently, using a new cultivation system called D-Root that prevents root illumination [12], a role for flavonols and ROS in root growth and meristematic activity has been uncovered. Flavonols seem to act as molecules that dynamically integrate auxin and cytokinin signaling with ROS function to control cell division and differentiation in the root meristem [13]. It should be highlighted that the natural environment for roots is darkness. Roots subjected to light undergo a burst of ROS and, subsequently, the accumulation of ROS scavengers that might mask and/or alter connections between different pathways operating in the root meristem. Bearing this in mind, to fully understand the role of ROS in meristem and QC maintenance, it will be important to eliminate, as much as possible, the effect of direct illumination of roots. It is possible that under dark-grown conditions, more interconnections between different pathways might be established. Another important question to be answered is how APP1 controls the level of *SCR* and *SHR*. Is it a direct control, or is it through the impact of ROS on hormonal signaling?

Although some questions await to be answered, an important and striking conclusion from this paper is that APP1-dependent ROS production is essential to maintain the stem cell niche in plants, preventing excess cell division in the QC and premature cell differentiation of the DSC.
